# Charge Transport and Phase Behavior of Imidazolium-Based Ionic Liquid Crystals from Fully Atomistic Simulations

**DOI:** 10.3390/ma11010064

**Published:** 2018-01-02

**Authors:** Michael J. Quevillon, Jonathan K. Whitmer

**Affiliations:** Department of Chemical and Biomolecular Engineering, University of Notre Dame, Notre Dame, IN 46556, USA; mquevill@nd.edu

**Keywords:** ionic liquids, liquid crystals, ionic liquid crystals, phase, charge transport

## Abstract

Ionic liquid crystals occupy an intriguing middle ground between room-temperature ionic liquids and mesostructured liquid crystals. Here, we examine a non-polarizable, fully atomistic model of the 1-alkyl-3-methylimidazolium nitrate family using molecular dynamics in the constant pressure–constant temperature ensemble. These materials exhibit a distinct “smectic” liquid phase, characterized by layers formed by the molecules, which separate the ionic and aliphatic moieties. In particular, we discuss the implications this layering may have for electrolyte applications.

## 1. Introduction

The intersection of the fields of ionic liquids and liquid crystals is a relatively new sector of materials science, but has garnered increased research in recent years [1,2,3,4], as ionic liquid crystals have begun to show promise as useful molecules in tunable conductive materials [5,6] or as greener solvents [7,8], for example. Liquid crystalline phases are of interest due to their innate mesostructure, arising from energetically- or entropically-driven alignment of molecules, or from phase separation arrested by molecular structure. This results in many different ordered phases [5,9,10,11], including multiple nematic, smectic, and columnar phases [10], among others. This wide variety of mesophases splits our traditional notion of a liquid, offering variable degrees and types of orientational and translational order without crystallization. Thermotropic liquid crystals tend to lose their partially ordered nature at high temperatures and form a completely disordered isotropic liquid phase [10], while lyotropic liquid crystals change phases at different concentrations, allowing for surfactant-like mesophases, such as micellar or bicontinuous cubic phases [12]. In solvent-free situations, liquid crystals commonly regarded as lyotropic behave thermotropically, exhibiting multiple interesting mesophases. Typical lyotropic liquid crystals are aqueous-solvated surfactants [13,14], with polar or charged head groups and aliphatic tails; the household soap sodium dodecyl sulfate (SDS) is one such example. There, the headgroup contains a relatively “hard” sulfate ion which associates with a compact sodium cation. When the solvent is removed, this results in a solid phase at room temperature [15]. If the headgroup is made bulkier, spreading out the charge and limiting close cation–anion contact, a liquid phase can be promoted at room temperature; this is the case in standard ionic liquids [16]. While most ionic liquids lack the extended alkyl chain similar to that found in SDS, thereby exhibiting only an isotropic liquid phase, addition of an extended tail can promote ordering via micro-phase separation, resulting in mesostructured ionic liquids, hence ionic liquid crystals.

Mesophases have long been observed in molten salt systems [17], but tuning the molecules to reach liquid crystal phases closer to room-temperature has been of more recent interest. Molecules studied at the intersection of these two fields are often based around imidazolium or viologen groups [18], with elongated alkyl tails attached to one or more ions. The counter-ion can be either a “simple” ionic liquid species (such as [PF_6_]^−^ or [BF_4_]^−^) or another ion with a long alkyl chain. The alkyl chain has a significant influence on the ordering properties observed, and the range of temperatures for which a given structure is stable, as it controls the relative energetic considerations for the formation of ordered ionic and apolar domains which drives ionic liquid crystal formation [19]. For a material to have partial long-range ordering and be considered an ionic liquid crystal, the threshold of alkyl chain length [20] varies with species and with counter-ion, indicating that a balance of electrostatic and van der Waals forces strongly influences the phases. The dominant order in ionic liquid crystal phases is one of lamellar or smectic character, typified by association and alignment of the long alkyl chains, with charged moieties also forming layers in the material [21]. These phases are highly reminiscent of the types of phases formed by lyotropic surfactants, though there is no added solvent. Different structures and degrees of ordering in ionic liquid crystals lead to material properties that can be harnessed for various uses. Ionic liquids have very promising applications as solvents [22,23], battery electrolytes [24,25], in CO_2_ sequestration [26,27], and in nonlinear-optical and photonic devices [28]. These properties can be further controlled when liquid crystal ordering is incorporated. As one example, smectic ionic liquid crystals used in battery electrolytes permit directionally-dependent ionic conductivity, much as is seen in block copolymer electrolytes [29].

Though significant benefits are present, these materials are highly nontrivial to synthesize. Hence, to guide synthetic chemists in determining useful targets, accurate computer simulations are of great utility. One can adopt many levels of description in simulations depending on the specificity with which one wants to sample configurational space. One such simple model utilizing Gay–Berne [30] and Lennard-Jones molecules is able to stabilize ionic liquid crystal phases by tuning the charge on each generic particle [31], however with some loss of generality. More accurate simulations are not without problems of their own, as force fields created for common ionic liquids [32,33,34,35,36,37] are not straightforward to translate into ionic liquid crystal systems. The naïve perspective of adding an alkyl tail to ionic species is not always straightforward. For simulations of ionic liquids, specialized force fields must be developed [38] to capture both thermodynamic and dynamic properties accurately. Even so, different choices for the interactions lead to an enthalpy–entropy trade-off, which is seen in many coarse-grained force fields [39]. The precise interplay of van der Waals and Coulombic interactions, perhaps including polarizable interactions [40], in determining macroscopic properties can lead to very accurate predictions for one molecule, but often lacks transferability to other models. While some coarse-graining techniques, such as Effective Force Coarse-Graining [35], have proven to be transferable for certain series of ionic liquid crystals [36], an all-atom approach is preferred for greater interoperability between molecules. All-atom approaches have seen limited applications to ionic liquid crystal systems, though a few notable investigations exist. Importantly, these are limited in scope, examining structural features in a narrow temperature range or are focused primarily on shorter alkyl chains.

Several generic force-field parameter sets, including GAFF [41], UFF [42], and OPLS [43] have been parameterized for a large number of common (and some less common) chemical groups, allowing for novel materials to be simulated without parameterizing a new force field for each. As a standard model for all-atom force fields, a modified OPLS-AA force field [33] provides generality to many series of common ionic liquids, with various lengths of alkyl side chains. The applicability of this all-atom force field to long molecules remains to be thoroughly studied, hence examining the effects of all-atom force fields in these systems is of chief interest in our investigation here. This force field contains many classes of molecules, hence its transferability to various types of ionic liquid crystals would be useful in studying this burgeoning field of molecular systems engineering.

**Figure 1:**
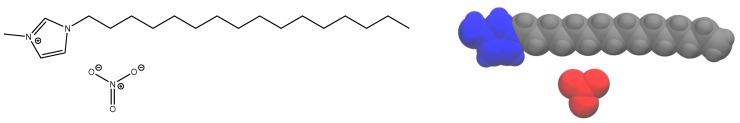
Chemical structure (**left**) and rendering of the atomistic model (**right**) of [C_16_MIm][NO_3_]. Colors in the rendered model signify the overall functional groups (blue: methyl imidazolium, gray: alkyl tail, red: nitrate).

We focus here on providing a detailed analysis of the phase behavior and dynamics using an all-atom model of 1-alkyl-3-methylimidazolium nitrate, compactly notated as [C_*n*_MIm][NO_3_] (see Figure 1 for an example structure). With long alkyl tails, this ionic liquid system forms liquid crystalline phases [21,44,45]. Since the charge groups for both species are relatively localized, the charge-containing domains can be structured in specific ways that have an anisotropy in their conductivity [46]. The extra ordering, when compared to isotropic ionic liquids, allows for greater tuning of interactions in the design of molecular media for catalysis [47] or other applications. The phase behavior of this particular system has not been studied in the ionic liquid crystal limit (long alkyl chains) from an all-atom simulation standpoint. In what follows, we will characterize the ordering of this molecular system using atomistic simulations and analysis of the dynamics within.

## 2. Methods

A series of 12-, 14-, and 16-carbon chain length 1-alkyl-3-methylimidazolium ([C_*n*_MIm]^+^) and nitrate ([NO_3_]^−^) ionic liquid crystals was simulated at constant atmospheric pressure (1 atm) in semi-isotropic or isotropic Parinello-Rahman simulation boxes with periodic boundary conditions. As discussed below, we performed temperature sweeps from 300 K to a maximum of 600 K using an ionic-liquid-modified OPLS-AA force field [33] in GROMACS 2016.3 [48], which contrasts with previous investigations using force fields such as CL&AP [49] or GAFF [50].

The energy of these systems is calculated from several separate terms, based on harmonic bonds and angles, Fourier series-based torsions, and nonbonded interactions including Coulombic and Lennard-Jones terms. The functional form for the potential energy, *E*, of OPLS [43] is shown in Equation (1), in terms of the length of bond, *r*, angle between bonds, θ, and torsional (or dihedral) angle, ϕ.

(1) $$\begin{array}{c} \begin{array}{cc} E= & \sum_{i\in \mathrm{bonds}}k_{r,i}{\left(r_{i}-r_{o,i}\right)}^{2}+\sum_{i\in \mathrm{angles}}k_{\theta ,i}{\left(\theta_{i}-\theta_{o,i}\right)}^{2}+\\ & \sum_{i\in \mathrm{torsions}}\frac{1}{2}\left[V_{1,i}\left(1+\cos\phi_{i}\right)+V_{2,i}\left(1+\cos2\phi_{i}\right)+V_{3,i}\left(1+\cos3\phi_{i}\right)+V_{4,i}\left(1+\cos4\phi_{i}\right)\right]+\\ & \sum_{i\in \mathrm{atoms}}\sum_{j>i}\frac{q_{i}q_{j}e^{2}}{r_{ij}}+4\varepsilon_{ij}\left[{\left(\frac{\sigma_{ij}}{r_{ij}}\right)}^{12}-{\left(\frac{\sigma_{ij}}{r_{ij}}\right)}^{6}\right] \end{array} \end{array}$$

The harmonic constants, $k_{r}$ and $k_{\theta }$, determine the strength of bonds and angles, respectively, around the minimum values of $r_{o}$ and $\theta_{o}$. The Fourier coefficients, $V_{n}$, define the torsional interaction strength within the molecules. The nonbonded interactions include the partial atomic charges, *q*, multiplied by the elementary charge, *e*, as well as the well depths, ε, and diameters, σ, of the Lennard-Jones potential for all particles.

Nitrate anions are considered to be rigid bodies, while the imidazolium-based species are able to fluctuate more freely. To allow appropriate relaxation of the layered phases, semi-isotropic pressure coupling is used to allow fluctuations within the layers (in the *x*- and *y*-directions) to decouple from the *z*-direction. To ensure proper representation of the fluid phase, an isotropic coupling is initiated after layers have melted. In these cases, the box dimensions from the previous temperature were used along with a constant pressure–constant temperature simulation to observe the isotropic liquid. Preliminary simulations using a fully charged model exhibited an overly stable crystal phase which was not observed to melt at temperatures less than 500 K. Thus, in order to predict material properties, the charges were uniformly scaled by 0.8, a modification commonly adopted in simulations of ionic liquids [34,51]. We found that this choice of scaling yielded behavior most consistent with prior studies [44,52,53]. However, the need to make such adjustments is indicative of inaccuracies in the force-field parameterization which necessitate eventual correction or reparameterization for ionic liquid crystalline systems. Importantly, we stress that while such a scaling may more accurately reproduce phase behavior and material dynamics, accurate resolution of charge transport and conductivity should retain q=1.0, and for these purposes the force balance should be corrected through other means [40].

**Figure 2:**
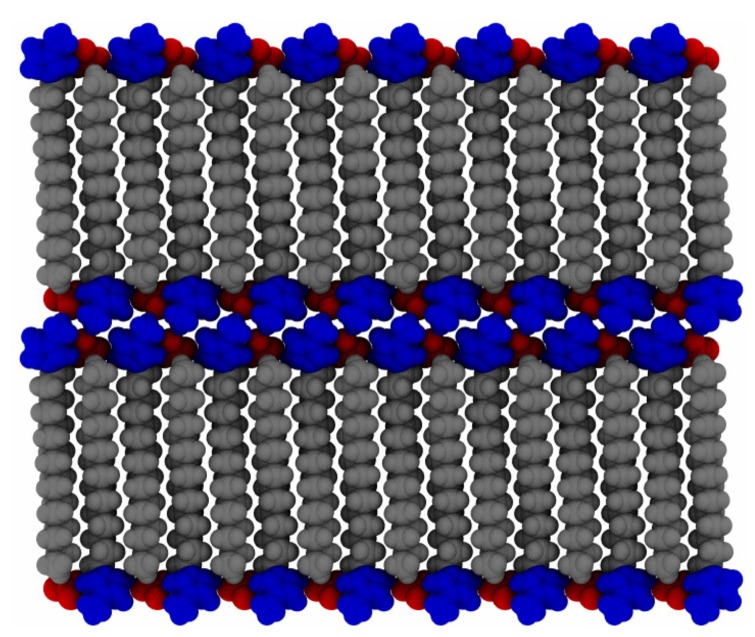
Initial configuration used in our simulation protocol. All of the alkyl tails are parallel, while neighboring molecules as a whole are antiparallel. Each dimension of each layer contains 16 evenly spaced molecules.

To probe a range of temperatures, the systems were set up in a proposed crystal configuration (see Figure 2) and equilibrated for 20 ns at 300 K, before increasing the temperature by discrete steps of 25 K until either the internal temperature reached 600 K or the material melted into an isotropic liquid. The latter condition was utilized for the n=12 simulations, as the semi-isotropic box exhibited instability within the “isotropic” phase. The initial configuration is created by minimizing a single pair of ions and then copying and rotating in each of the Cartesian axes of the molecule, to acquire a unit cell of 8 ion pairs, with a point group of $D_{2d}$. This is then copied and translationally arranged into 2 layers of 16×16 molecules, for a total of 512 ion pairs. This does not necessarily correspond to an equilibrated structure of the full system, particularly as multiple potential ionic arrangements are possible in crystalline states of ionic liquid crystal species [54], but merely provides a good starting point for investigations of layered structures. A “pre-conditioning” simulation of 5 ns at the starting temperature allows the hypothetical crystalline state (at 300 K) to equilibrate the system partially before production simulations. The systems were allowed to equilibrate for 20 ns, during the initial temperature ramp, before increasing to the next temperature, in series. Data from each temperature step were generated starting from the final configuration of the previous (lower) temperature, to allow for a more gradual thermal relaxation. Then, each temperature was allowed to equilibrate for an additional 280 ns, in parallel, for a total simulation time of 300 ns at each temperature. This ramping scheme followed from a coarse-grained simulation of similar molecules [21] using discrete changes in temperature, followed by further equilibration.

All simulations were run with a timestep of 1 fs, using a stochastic dynamics integrator (“sd” in GROMACS) and a Parrinello-Rahman barostat, with time constants of 2 ps and 1 ps, respectively. The compressibility was assumed to be that of water, or $4.5\times 10^{-5}$ bar. These parameters are standard molecular dynamics values for fully atomistic simulations. As the initial configuration was set up to be somewhat unphysical, the box sizes quickly converged on more physical values. Since the system is symmetric in the *x*- and *y*-directions, the aspect ratio of these dimensions was set to unity, while the *z*-direction’s initial value was based on the length of the molecule when equilibrated in vacuum.

## 3. Results

**Figure 3:**
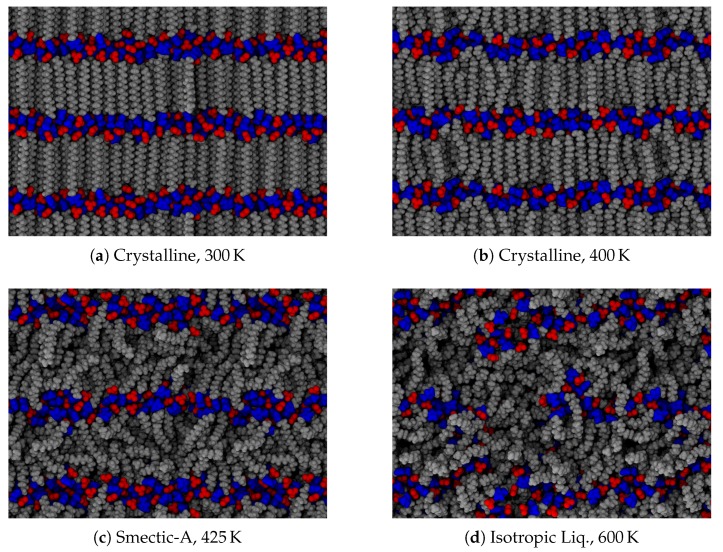
Observed phases in the ionic liquid crystal material, [C_16_MIm][NO_3_]. Typical configurations are presented from temperatures ranging between (**a**) 300 K and (**d**) 600 K. Frustrations within the crystal are visible in (**a**), as some aliphatic tails are bent within the layer and anions exhibit irregular placement. Intermediate temperatures illustrated in (**b**,**c**) depict the system on either side of the crystal–smectic phase transition. The isotropic liquid still exhibits segregation, but the layers have lost their long-range order, as evidenced by (**d**).

As noted in the introduction, a typical material will undergo a phase change from a crystalline solid to an isotropic liquid, often observed through a sharp change in its volume. Liquid crystals, however, exhibit additional liquid phases of varying degrees of order. Upon heating our crystalline initial configurations, both ionic groups and aliphatic tails between the layers of charged groups can begin to move more freely among each other. As the energetic effects holding each layer together are of different origin (van der Waals for the tails and Coulombic for the ionic groups), the different regions may have different effective melting temperatures, leading to further splitting of the observed phases. This is the case here, as depicted in Figure 3. At low temperatures, the layered structure of the crystal prevails, though thermal fluctuations cause some frustration within the crystal. In our system, this corresponds to disordering of the ionic placements, and some slight melting of the aliphatic tails (see Figure 3a), though there is still a strong degree of translational and orientational order, as will be discussed later. As this system is heated, the order among the tails begins to relax and the ionic domain becomes more disordered (see Figure 3b). Though we will show later this state exhibits properties suggesting it is a distinct phase, a clear signature is not visible in the global thermodynamic variables we calculate, and hence we label this state also as a crystal. With additional heat, tail conformations melt significantly, though the layered structure is retained, forming a smectic-A phase [21]. Here, layers begin to pull slightly away from each other, and the spacing of layers is broadened as evidenced in Figure 3c. In molecules with a short alkyl chain, smectic phases are not observed, making such phases indicative of the class of ionic liquid crystals, though metastable phases which may include smectic-like order have also been reported [50]. The disorder between layers does not permeate through the layers, which means that the overall layered structure of the system does not differ between the crystalline and smectic phases. Nevertheless, there is a slight relaxation of the layer spacing in the smectic-A phase, since the alkyl chains are no longer optimally packed. Further heating of the system removes the layered structure (Figure 3d). While we refer to this as an isotropic liquid, there are clearly still some domains of charge segregation, indicative of the charged clusters often observed in simulations of ionic liquids [55,56].

**Figure 4:**
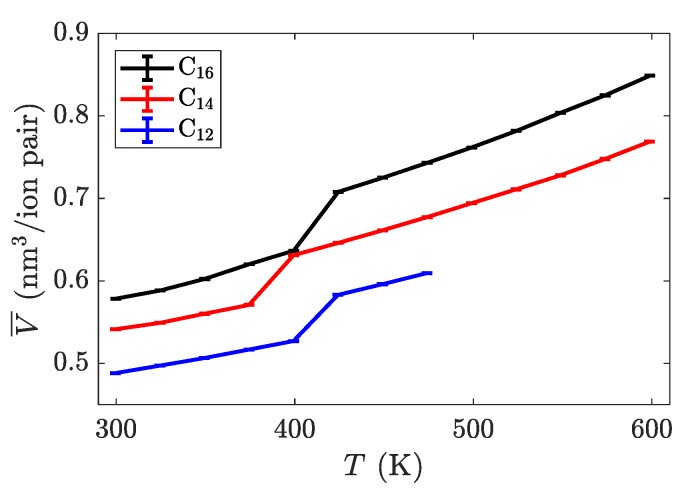
Intrinsic volume per ion pair as a function of temperature for three different alkyl chain lengths. The most dramatic jump in volume is between the crystalline and smectic phases. The rightmost point on each line is where the material melted into an isotropic liquid. Transition temperatures are summarized in Table 1.

**Figure 5:**
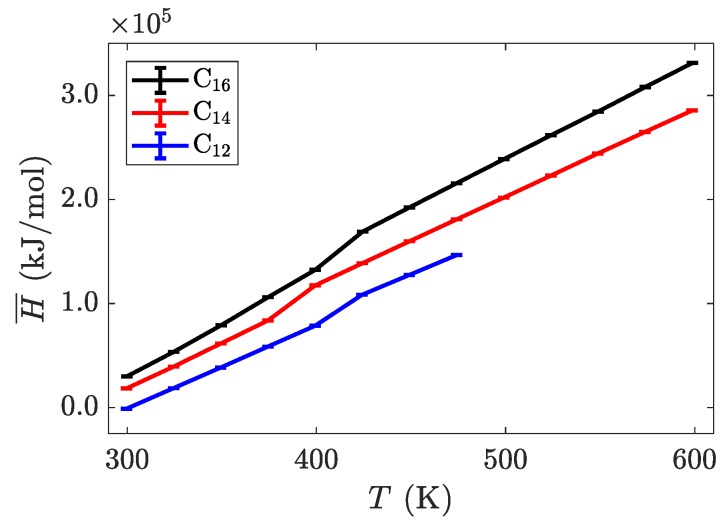
Intrinsic enthalpy as a function of temperature for three different alkyl chain lengths. The phase transitions are less pronounced in this system-wide property, but can be identified by the changes in slope of each line.

The characterization of phase transitions may be done in multiple ways. For simulations of ionic liquid crystals, the intrinsic property of volume per ion pair (equivalent to an inverse number density) is often useful [21], as it can characterize significant jumps in the effective size of molecules, indicative of increased disorder. Within a single phase, volume as a function of temperature is close to linear, but a phase change is observed as a discontinuity or jump in this line. As Figure 4 shows, the transition between the crystalline and the smectic-A phase is seen quite easily for all three homologues. This is expected to have a significant signature, due to the change in layer spacing during this transition (see Figure 3c). The transition between the smectic-A phase and the isotropic liquid is less pronounced, but is confirmed through visualization or by characterizing the aliphatic ordering. These transitions are first order, and should be characterized by both a latent heat signature and a change in the heat capacity before and after the transition. Albeit, the phase transitions are less pronounced when examining this quantity, partially due to the fairly large temperature steps ( 25 K) utilized in our study (see Figure 5).

To better understand the ordering of liquid crystalline phases, we compute the orientational order parameter *S* by defining a nematic director for each molecule from the axis with the smallest moment of inertia, $\hat{n}$. The Q-tensor [57] was subsequently calculated via Equation (2).

(2) $$Q\left(t\right)=\frac{3}{2N}\sum_{i=1}^{N}\left({\hat{n}}_{i}\left(t\right)⊗{\hat{n}}_{i}\left(t\right)-\frac{1}{3}I\right)$$

**Figure 6:**
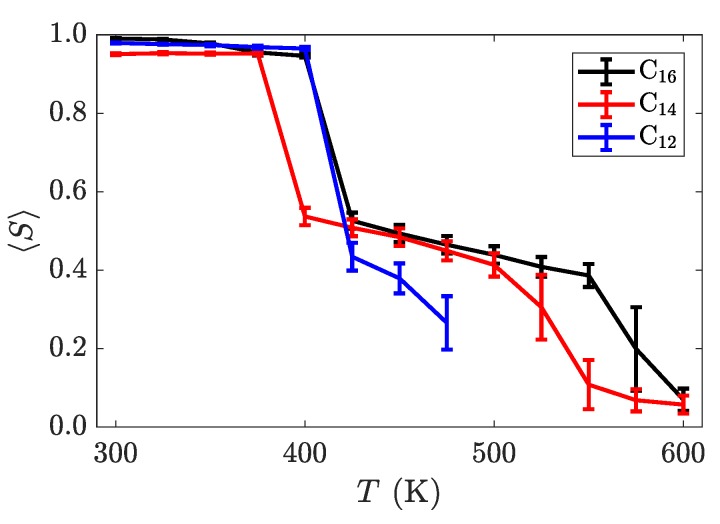
Nematic order parameter as a function of temperature for three different alkyl chain lengths, averaged over the last 200 ns of the trajectory. Two distinct phase transitions can be observed a discontinuous jumps in this order parameter. The error bars are larger on temperatures near transition points, as the systems fluctuate more there, thus introducing more variance into trajectory-averaged properties.

The orientational order parameter, *S*, is obtained from the largest positive eigenvalue of the characteristic equation of Q(t). The results, time-averaged for the last 200 ns of each temperature in the sweep, are summarized in Figure 6. Interestingly, this graph demonstrates a non-monotonicity in transition temperature with alkyl tail length, with a broadened smectic-A phase also appearing for *n* = 14 and *n* = 16. Though a more thorough investigation is required to understand the exact cause for this behavior, we hypothesize that it might involve a trade-off between crystal orderings dominated by electrostatic and van der Waals interactions, as inter-layer Coulombic interactions should be felt more strongly between layers when the aliphatic tails are short, while van der Waals interactions would have increased influence as the tails grow. Such behavior might be used to tune the position of the crystalline to smectic transition through the incorporations of softer ions or more structured tails with stronger van der Waals interactions.

While the trends in the transition temperatures (listed in Table 1) of these atomistic simulations are similar to experimental measurements [19], the exact numbers differ. These differences are also seen in simulations using transferable coarse-grained models [21]. This is indicative of a more permeating issue concerning molecular force fields; standard models are not able to capture the entire behavior of this class of materials. Often, the same procedure can yield accurate results for smaller homologues, but miss out on the behavior of longer aliphatic chains, as is observed [58] for long homologues of [C_*n*_MIm][PF_6_].

In fact, when compared to experimental data [19], these transition temperatures are more comparable with [C_*n*_MIm][Cl], which has a much smaller chloride anion, instead of the bulkier nitrate anion. The size of the chloride ion allows for a more focused effect of Coulombic interactions, as the charge is not spread over a large area. This, in turn, raises the temperature at which phase transitions occur, with more thermal energy required to overcome the Coulombic attraction between ions. This may not be the only effect causing the large gap separating computational and experimental transition temperature measurements. In particular, the polarizability inherent in the molecular system is poorly represented by the constant charges imposed within our model. This could potentially be addressed by adding polarizable sites [59] to allow for variation in the distribution of charge.

While the data above were gathered from increasing temperature sweeps, we note that the smectic phase was also observed to spontaneously assemble from initially isotropic conditions above the melting transition. Simulations were performed at low temperatures starting from random configurations to ensure that our initial crystalline configuration was feasible for these systems. This confirmed that our system was equilibrated, as the phase was the same whether approached from lower or higher temperatures. If the layered structures were only metastable, these simulations would stay isotropic, instead of assembling into mesostructures spontaneously. It is possible that some hysteresis exists close to the melting temperatures due to metastability of the ordering behavior, as has been observed in recent simulations of ionic liquids and liquid crystals [50].

**Figure 7:**
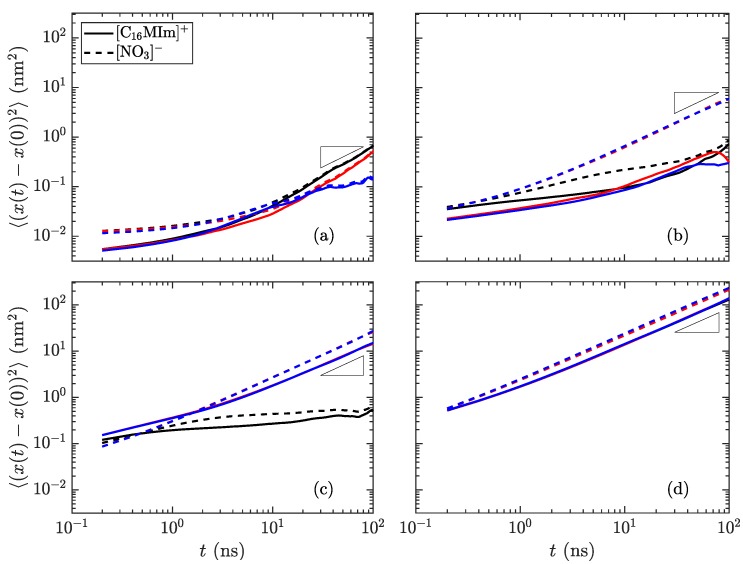
Mean-squared displacement of the species in [C_16_MIm][NO_3_] as a function of time at temperatures of (**a**) 300 K in the crystalline state, (**b**) 400 K just before the transition to smectic, (**c**) 425 K in the smectic-A phase, and (**d**) 600 K isotropic liquid. Reference slopes of 1, indicated as small triangles, help to identify the linear diffusive regime. The colors signify the dimension (red: *x*, blue: *y*, black: *z*); the solid lines are for the long cations and the dotted lines are for the nitrate anions. The most distinctive difference is the divergence of the *z*-direction from the *x*- and *y*-directions in the smectic-A phase, showing that transport within a layer is much simpler than through the layers. (Notes: (**c**) The *x*- and *y*-directions are nearly overlapping. (**d**) All directions nearly collapse, indicating an isotropic phase).

Another method of identifying phases is to monitor the mobility of molecules as the simulations progress. In the lower-temperature, crystalline regime, all atoms should have low mobility. As the material transitions to the smectic phase, the charge groups (the aromatic imidazolium rings and the anions) are fixed into thin layers, while the alkyl tails are free to move within their layer. The final phase transition sees mobility of all atoms of all species. Figure 7 shows the difference in mobility for select temperatures in these simulations. Near the transition temperature between crystalline and smectic phases, the long cations are still limited in their crystalline mobility, but the smaller nitrate ions are able to move much more freely, as observed as a divergence between the in-plane mobility of the nitrate anions and imidazolium cations in Figure 7b, which, though observed to contain increased disorder within the ionic layers in Figure 3b, is still classified as the crystalline phase. This behavior near the transition temperature may signify an interesting design space, where the different ionic species have highly divergent mobilities.

The different mobility signatures of the various phases allow for greater tunability in ion transport. For the phases unique to liquid crystals, the anisotropy of movement can control the transport of smaller species through the charged domains, as any small ions will remain confined within these regions rather than traversing between layers. By going to the low-temperature limit, all species have restricted mobility; the high-temperature limit allows for unhindered motion of all species. It is within these limits where the liquid crystalline properties are useful in determining the material order and directional conductivity for small charged species. These types of material show promise as having intrinsic methods for controlling ion (and hence charge) transport, which could prove useful in applications where the direction of ion transport is of prime importance. In particular, layered domains which form bulk smectic phases are likely to form conductive contacts with other layers at grain boundaries within a sample quenched into a solid or glass-like state, increasing the flow of charge carriers through the medium. Such topics are clear motivators for future work examining quantitatively the interplay of charged and apolar domains in these materials, and their influence on ion conduction between common cathode and anode materials.

**Figure 8:**
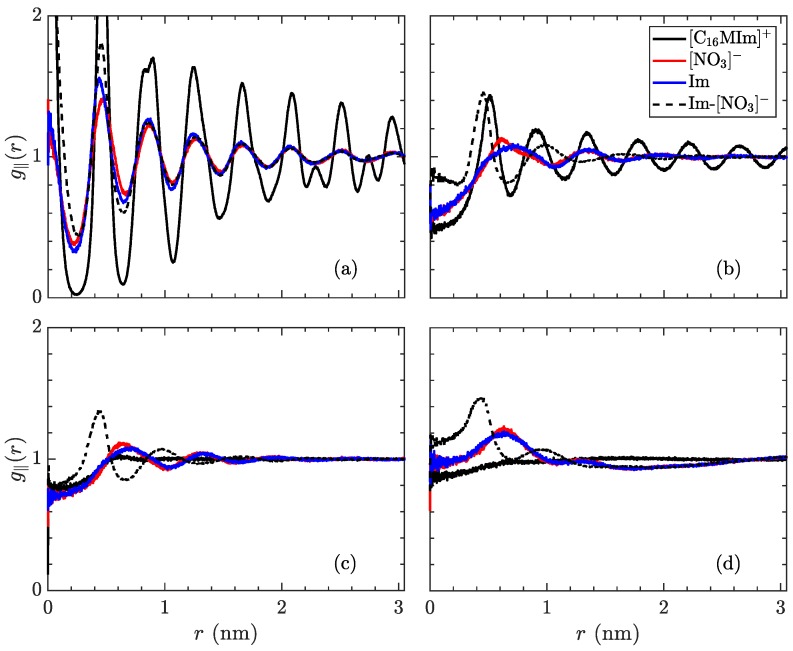
In-plane radial distribution function, $g_{∥}\left(r\right)$ of the species in [C_16_MIm][NO_3_], trajectory-averaged over the last 200 ns, at temperatures of (**a**) 300 K in the crystalline state, (**b**) 400 K just before the transition to the smectic-A state, (**c**) 425 K in the smectic-A phase, and (**d**) 600 K isotropic liquid. The solid lines signify distribution of a species relative to its own type (black: [C_16_MIm]^+^, red: [NO_3_]^−^, blue: imidazolyl ring) while the dotted line denotes placement of [NO_3_]^−^ ions in relation to imidazolyl rings. The first phase transition is characterized by the alkyl tails losing almost all order within the xy-plane. The phase transition to the isotropic liquid loses almost all structure, except for pairing of the opposite charges.

The radial distribution function can also be indicative of structure in ionic liquid crystal phases. Due to the layered nature of these simulations, the three-dimensional form of g(r) misses several properties of the low-temperature phases. Hence, the in-plane radial distribution function, $g_{∥}\left(r\right)$, is more relevant for this system, notwithstanding its collapsing of the *z*-direction. In an ideal crystal, this function will show sharp peaks at the locations of each molecule’s nearest neighbors. However, introducing a nonzero temperature will spread out these peaks, as thermal fluctuations disrupt the perfect ordering. On the opposite side of the temperature range, an isotropic liquid will show no preference for order, therefore the averaged density around a certain molecule will be uniform. These profiles are shown in Figure 8, for the same temperatures as previous figures.

As with the mean-squared displacement, an interesting change occurs before the crystalline–smectic transition, as the charged moieties begin to lose their long-range order, while the longer molecules retain more order. Coupled with the observations regarding ionic mobility, this is very suggestive of an intermediate phase appearing in advance of the smectic-A phase, where the ionic layers are melted, but the aliphatic tails retain crystal structure. As no signature is observed in our measurements of volume per ion pair and intrinsic enthalpy, there is insufficient evidence to state a phase transition exists, though more detailed measurements of the thermodynamic variables in this region should be able to detect if this is a transient condition or a separate equilibrium phase. After transitioning to the smectic-A phase, the centers of the charged groups continue to exhibit ordering, as the planes of ionic domains are still intact, but the alkyl tails are more disordered within the aliphatic domains. As previously mentioned, the high temperature smectic–isotropic transition only refers to melting of the layers and does not result in a fully isotropic liquid, as evidenced by the short-range structure of ionic groups, quickly decaying after a few nearest neighbors.

## 4. Discussion and Conclusions

A fully atomistic investigation into the phase behavior of an imidazolium-based ionic liquid crystal series was performed. At these lengths of alkyl side chains, three distinct phases are observed for temperatures between 300 K and 600 K: crystalline, smectic-A, and an isotropic liquid. The length of the alkyl chain plays an important role in defining the conditions under which these molecular systems change phases. Using an all-atom model allows for greater transferability at a small computational cost, since many ionic liquid species were parameterized with the goal of interoperability between species.

While the general trends of the transition temperatures are correct, there are differences between this work and previous investigations [21]. The main source of disagreement is the type of model used: all-atom versus coarse-grained. Prior to this work, fully atomistic simulations of this degree have not been pursued. In addition, there may be added entropy due to the number of additional atoms to simulated. However, the trends for these long molecules extrapolate to suggest that [C_10_MIm][NO_3_] does not have liquid crystal-like behavior, which is supported by previous investigations [19]. Importantly, we also observe an interesting dynamic behavior just prior to the crystalline–smectic transition which could be the hallmark of another phase or an intriguing metastable state; more detailed study is necessary to understand this state completely. While at even higher temperatures, there may be several non-layered mesophases that these systems exhibit; we do not observe any significant thermodynamic or structural changes aside from the crystalline–smectic and smectic–isotropic transitions for the temperatures studied here. Thus, due to the high temperatures needed to observe these phases, the characterization of these phases is likely of less practical interest.

Materials of this class may prove useful in various applications, such as forming a structured medium for batteries where the conductivity in the different directions can be tuned. At the specified temperatures, the charged moieties are confined to thin layers, with essentially no transfer between layers, which directs the mobility of ions. While the motions of this material were characterized here, the addition of other ions will require further investigations into diffusive transport through these materials.

## Figures and Tables

**Table 1:** Observed transition temperatures between the various phases for this series of ionic liquid crystals. Lee, et al. [19] did not observe any smectic phase for [C_12_MIm][NO_3_], but observed the isotropic phase above 318 K. As a function of alkyl tail length, the lower transition temperature exhibits a slight non-monotonicity for these species.

Species	Transition	Temperature, K		
		This Work	Experimental [19]	Coarse-Grained [21]
C_12_	Crystal–Smectic-A	425	-	-
	Smectic-A–Isotropic	475	-	-
C_14_	Crystal–Smectic-A	400	336	-
	Smectic-A–Isotropic	550	356	-
C_16_	Crystal–Smectic-A	425	339	500
	Smectic-A–Isotropic	575	404	560

## Abbreviations

The following abbreviations are used in this manuscript:

[C_*n*_MIm]^+^	1-alkyl-3-methylimidazolium
[NO_3_]^−^	nitrate
[PF_6_]^−^	hexafluorophosphate
[BF_4_]^−^	tetrafluoroborate
SDS	sodium dodecyl sulfate
