# Correction: Quevillon, M.J., et al. Charge Transport and Phase Behavior of Imidazolium-Based Ionic Liquid Crystals from Fully Atomistic Simulations. *Materials* 2018, *11*, 64

**DOI:** 10.3390/ma14010120

**Published:** 2020-12-30

**Authors:** Michael J. Quevillon, Jonathan K. Whitmer

**Affiliations:** Department of Chemical and Biomolecular Engineering, University of Notre Dame, Notre Dame, IN 46556, USA; mquevill@nd.edu

**Keywords:** ionic liquids, liquid crystals, ionic liquid crystals, phase, charge transport

## 1. Introduction

In our publication [1], we performed classical molecular dynamics simulations of a system of ionic liquid crystals using a transferable OPLS-AA force field [2] specifically developed for ionic liquids. In performing subsequent research, we became aware of a paper [3] performed by the same authors as reference [2], which updates some of the force field parameters for molecular dynamics simulations. However, the functional form of the torsional angles in the newer paper [3] was listed in a different form than was used in the original paper of the force field [2]. This lead us to review the force field that we had implemented in our study [1] and to determine if this had had significant quantitative or qualitative effects.

With corrected functional form in the subsequent publication [3], the authors wish to make corrections to our publication [1], as well as replace the affected figures. All references to the original force field have been updated to include the subsequent publication.

Additionally, we correct an experimental comparison within Table 1, which used data on [C${}_{n}$Im][NO${}_{3}$] rather than [C${}_{n}$MIm][NO${}_{3}$].

## 2. Text Corrections

The functional form of the OPLS-AA force field is corrected below. Replace

(1) $$\begin{array}{c} \begin{array}{cc} E=\; & \sum_{i\in \mathrm{bonds}}k_{r,i}{\left(r_{i}-r_{o,i}\right)}^{2}+\sum_{i\in \mathrm{angles}}k_{\theta ,i}{\left(\theta_{i}-\theta_{o,i}\right)}^{2}+\\ \; & \sum_{i\in \mathrm{torsions}}\frac{1}{2}\left[V_{1,i}\left(1+\cos\phi_{i}\right)+V_{2,i}\left(1+\cos2\phi_{i}\right)+V_{3,i}\left(1+\cos3\phi_{i}\right)+V_{4,i}\left(1+\cos4\phi_{i}\right)\right]+\\ \; & \sum_{i\in \mathrm{atoms}}\sum_{j>i}\frac{q_{i}q_{j}e^{2}}{r_{ij}}+4\varepsilon_{ij}\left[{\left(\frac{\sigma_{ij}}{r_{ij}}\right)}^{12}-{\left(\frac{\sigma_{ij}}{r_{ij}}\right)}^{6}\right] \end{array} \end{array}$$

with

(1) $$\begin{array}{c} \begin{array}{cc} E=\; & \sum_{i\in \mathrm{bonds}}\frac{1}{2}k_{r,i}{\left(r_{i}-r_{o,i}\right)}^{2}+\sum_{i\in \mathrm{angles}}\frac{1}{2}k_{\theta ,i}{\left(\theta_{i}-\theta_{o,i}\right)}^{2}+\\ \; & \sum_{i\in \mathrm{torsions}}\frac{1}{2}\left[V_{1,i}\left(1+\cos\phi_{i}\right)+V_{2,i}\left(1-\cos2\phi_{i}\right)+V_{3,i}\left(1+\cos3\phi_{i}\right)+V_{4,i}\left(1-\cos4\phi_{i}\right)\right]+\\ \; & \sum_{i\in \mathrm{atoms}}\sum_{j>i}\frac{q_{i}q_{j}e^{2}}{r_{ij}}+4\varepsilon_{ij}\left[{\left(\frac{\sigma_{ij}}{r_{ij}}\right)}^{12}-{\left(\frac{\sigma_{ij}}{r_{ij}}\right)}^{6}\right] \end{array} \end{array}$$

In addition, with the corrected force field, the nitrate anions are no longer rigid [3], so remove the following sentence from line 110:

- Nitrate anions are considered to be rigid bodies, while the imidazolium-based species are able to fluctuate more freely.

With the adjusted force field [3], there were certain aspects of the simulation protocol that were slightly adjusted. Replace the following sentences, starting at line 126,

- To probe a range of temperatures, the systems were set up in a proposed crystal configuration (see Figure 2) and equilibrated for 20 ns at 300 K, before increasing the temperature by discrete steps of 25 K until either the internal temperature is 600 K or the material melts into an isotropic liquid. The latter condition was utilized for the n=12 simulations, as the semi-isotropic box exhibited instability within the “isotropic” phase.

with

- To probe a range of temperatures, the systems were set up in a proposed crystal configuration (see Figure 2) and equilibrated for 20 ns at 300 K, before increasing the temperature by discrete steps of 25 K up to 600 K.

Add the following comment on the low-temperature crystal configurations at line 158,

- Despite starting from a parallel initial configuration, these systems adopted a tilted, chevron-like ordering upon equilibration. This tilting is consistent with other simulations [4], but the alternating directions of tilt is an effect of our chosen initial configuration. The simplest way for the system to transform into a tilted configuration is by moving the central ionic layer in one direction as a whole, which induces the chevron-like ordering observed here.

Replace the beginning of the sentence starting at line 158,

- Though we will show later this state exhibits properties suggesting it is a distinct phase …

with

- Though we will show later this state exhibits structural and dynamic properties suggesting it is a distinct phase …

One important difference between the prior results and those with the corrected force field is addressed below.

Replace the following sentences, starting at line 198,

- Interestingly, this graph demonstrates a non-monotonicity in transition temperature with alkyl tail length, with a broadened smectic-A phase also appearing for n=14 and n=16. Though a more thorough investigation is required to understand the exact cause for this behavior, we hypothesize that it might involve a trade-off between crystal orderings dominated by electrostatic and van der Waals interactions, as inter-layer Coulombic interactions should be felt more strongly between layers when the aliphatic tails are short, while van der Waals interactions would have increased influence as the tails grow.

with

- By increasing the length of the alkyl side chain, the relative influence of the inter-layer Coulombic interactions and the van der Waals interactions stabilizes the smectic-A phases, thereby broadening the range of temperatures where the smectic-A is observed.

The long-range ordering behavior somewhat changed with the corrected force field [3], which required altered commentary. In line 266, replace

- As with the mean-squared displacement, an interesting change occurs before the crystalline–smectic transition, as the charged moieties begin to lose their long-range order, while the longer molecules retain more order.

with

- As with the mean-squared displacement, an interesting change occurs before the crystalline–smectic transition, where the charged moieties (both [NO_3_]^−^ and [C_14_MIm]^+^) begin to exhibit a more smoothed radial distribution function than the aliphatic tails.

## 3. Figure Corrections

Replace

**Figure 3:**
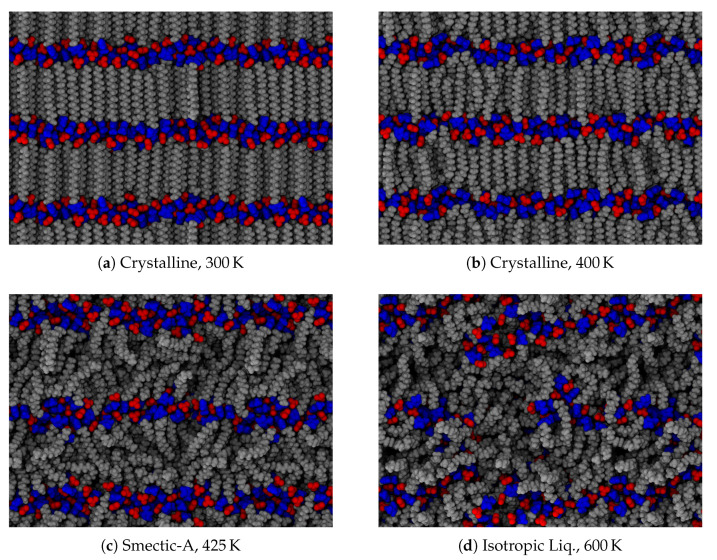
Observed phases in the ionic liquid crystal material, [C_16_MIm][NO_3_]. Typical configurations are presented from temperatures ranging between (**a**) 300 K and (**d**) 600 K. Frustrations within the crystal are visible in (**a**), as some aliphatic tails are bent within the layer and anions exhibit irregular placement. Intermediate temperatures illustrated in (**b**,**c**) depict the system on either side of the crystal–smectic phase transition. The isotropic liquid still exhibits segregation, but the layers have lost their long-range order, as evidenced by (**d**).

with

**Figure 3:**
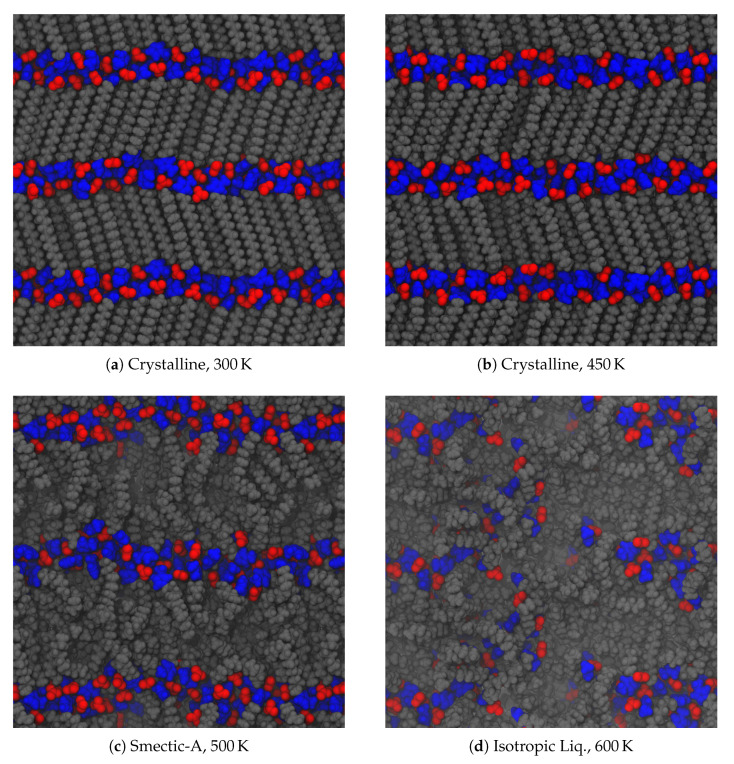
Observed phases in the ionic liquid crystal material, [C_16_MIm][NO_3_]. Typical configurations are presented from temperatures ranging between (**a**) 300 K and (**d**) 600 K. The tilted layers in (**a**) are consistent with other models [4], though the alternating direction of tilt is most likely due to our simulations’ proposed initial configurations. Frustrations within the crystal are visible in (**a**), as some aliphatic tails are bent within the layer and anions exhibit irregular placement. Intermediate temperatures illustrated in (**b**,**c**) depict the system on either side of the crystal–smectic phase transition. The isotropic liquid still exhibits segregation, but the layers have lost their long-range order, as evidenced by (**d**).

Replace

**Figure 4:**
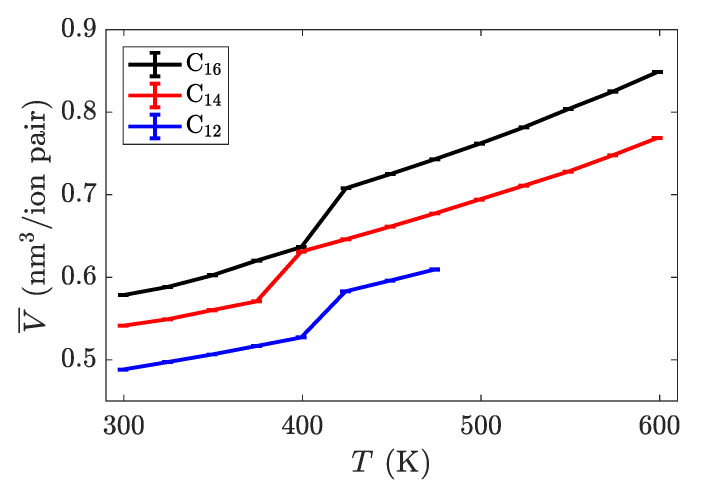
Intrinsic volume per ion pair as a function of temperature for three different alkyl chain lengths. The most dramatic jump in volume is between the crystalline and smectic phases. The rightmost point on each line is where the material melted into an isotropic liquid. Transition temperatures are summarized in Table 1.

with

**Figure 4:**
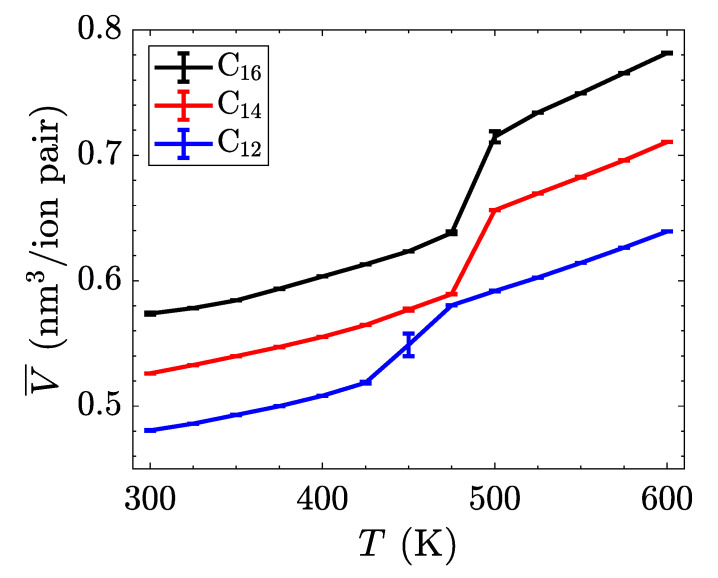
Intrinsic volume per ion pair as a function of temperature for three different alkyl chain lengths. The most dramatic jump in volume is between the crystalline and smectic phases. The transition between the smectic and isotropic phases is not apparent in this quantity. Transition temperatures are summarized in Table 1.

Replace

**Figure 5:**
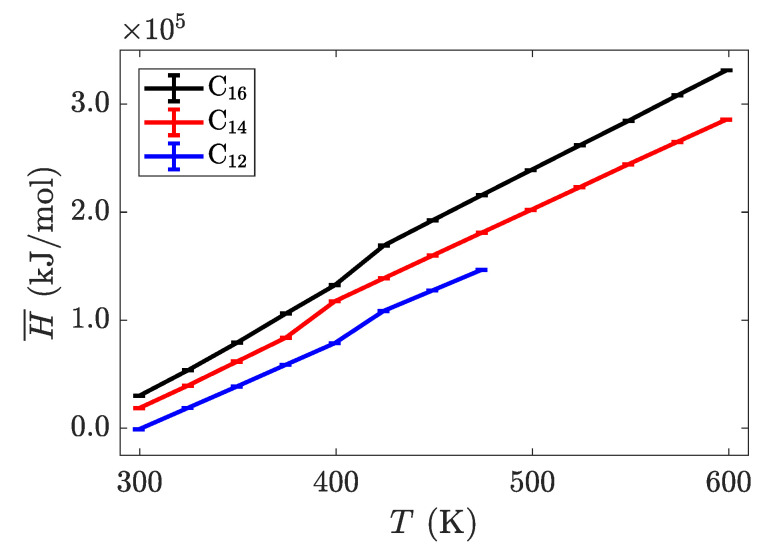
Intrinsic enthalpy as a function of temperature for three different alkyl chain lengths. The phase transitions are less pronounced in this system-wide property, but can be identified by the changes in slope of each line.

with

**Figure 5:**
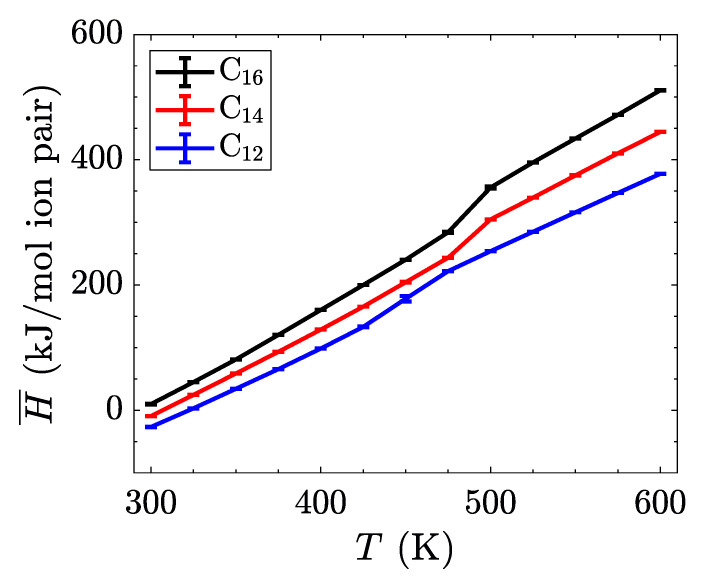
Intrinsic enthalpy as a function of temperature for three different alkyl chain lengths. The phase transitions are less pronounced in this system-wide property, but can be identified by the changes in slope of each line.

Replace

**Figure 6:**
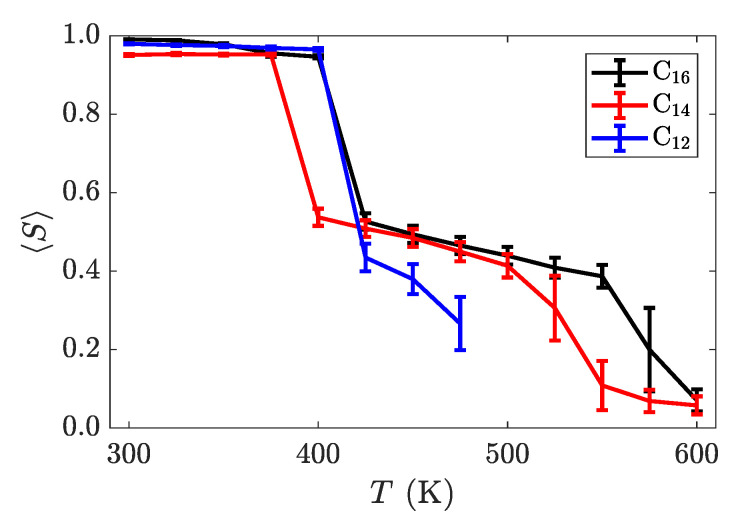
Nematic order parameter as a function of temperature for three different alkyl chain lengths, averaged over the last 200 ns of the trajectory. Two distinct phase transitions can be observed a discontinuous jumps in this order parameter. The error bars are larger on temperatures near transition points, as the systems fluctuate more there, thus introducing more variance into trajectory-averaged properties.

with

**Figure 6:**
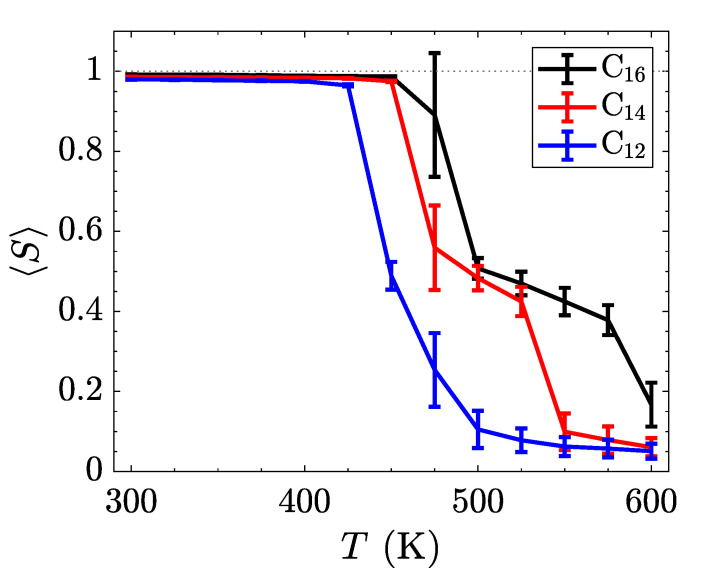
Nematic order parameter as a function of temperature for three different alkyl chain lengths, averaged over the last 200 ns of the trajectory. The layers are calculated separately and averaged, due to the opposite tilting directions in the crystal phase. Two distinct phase transitions can be observed as discontinuous jumps in this order parameter. The error bars are larger on temperatures near transition points, as the systems fluctuate more there, thus introducing more variance into trajectory-averaged properties.

Replace

**Table 1:** Observed transition temperatures between the various phases for this series of ionic liquid crystals. Lee, et al. [5] did not observe any smectic phase for [C_12_MIm][NO_3_], but observed the isotropic phase above 318 K. As a function of alkyl tail length, the lower transition temperature exhibits a slight non-monotonicity for these species.

Species	Transition	Temperature, K		
		This Work	Experimental [5]	Coarse-Grained [6]
C_12_	Crystal–Smectic-A	425	-	-
	Smectic-A–Isotropic	475	-	-
C_14_	Crystal–Smectic-A	400	336	-
	Smectic-A–Isotropic	550	356	-
C_16_	Crystal–Smectic-A	425	339	500
	Smectic-A–Isotropic	575	404	560

with

**Table 1:** Observed transition temperatures (upper bounds) between the various phases for this series of ionic liquid crystals. Guillet et al. [7] did not observe any smectic phase for [C_12_MIm][NO_3_], but observed the isotropic phase above 302 K. As a function of alkyl tail length, the trends of transition temperatures are consistent with experimental results [7].

Species	Transition	Temperature, K		
		This Work	Experimental [7]	Coarse-Grained [6]
C_12_	Crystal–Smectic-A	450	-	-
	Smectic-A–Isotropic	500	-	-
C_14_	Crystal–Smectic-A	475	315	-
	Smectic-A–Isotropic	550	401	-
C_16_	Crystal–Smectic-A	500	320	505
	Smectic-A–Isotropic	575	457	560

Replace

**Figure 7:**
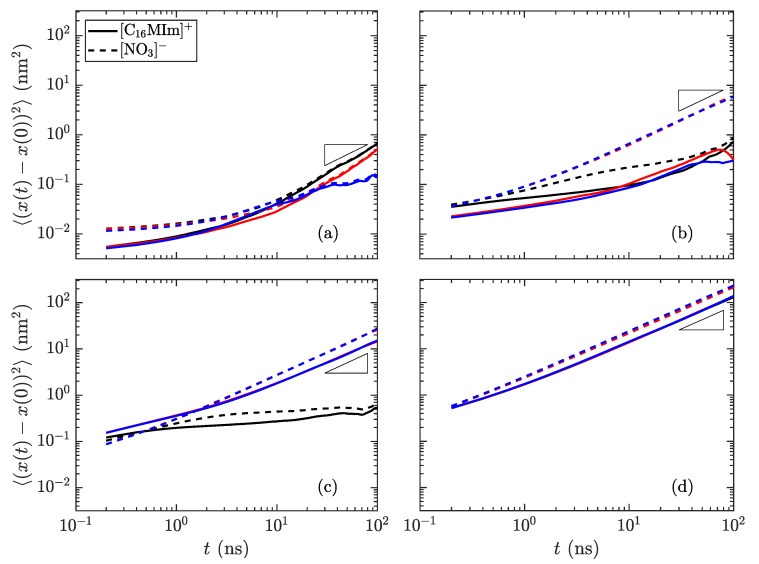
Mean-squared displacement of the species in [C_16_MIm][NO_3_] as a function of time at temperatures of (**a**) 300 K in the crystalline state, (**b**) 400 K just before the transition to smectic, (**c**) 425 K in the smectic-A phase, and (**d**) 600 K isotropic liquid. Reference slopes of 1, indicated as small triangles, help to identify the linear diffusive regime. The colors signify the dimension (red: *x*, blue: *y*, black: *z*); the solid lines are for the long cations and the dotted lines are for the nitrate anions. The most distinctive difference is the divergence of the *z*-direction from the *x*- and *y*-directions in the smectic-A phase, showing that transport within a layer is much simpler than *through* the layers. (Notes: (**c**) The *x*- and *y*-directions are nearly overlapping. (**d**) All directions nearly collapse, indicating an isotropic phase.)

with

**Figure 7:**
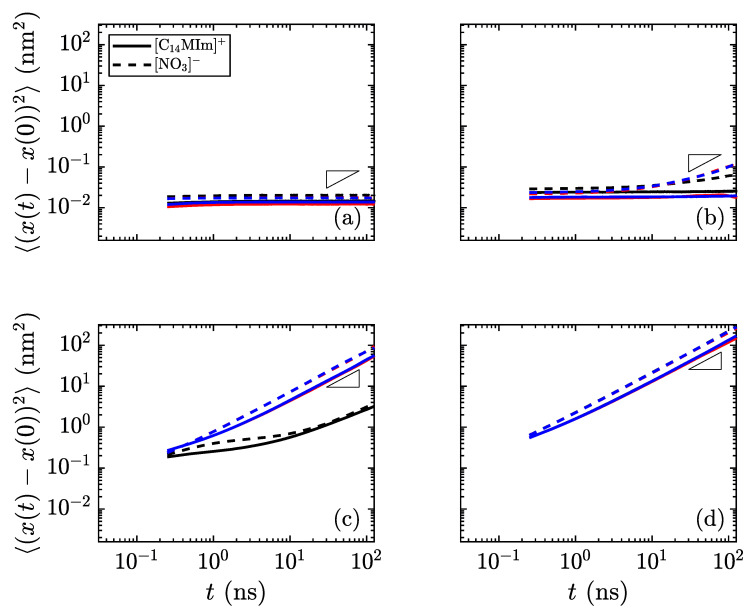
Mean-squared displacement of the species in [C_14_MIm][NO_3_] as a function of time at temperatures of (**a**) 400 K in the crystalline state, (**b**) 450 K just before the transition to smectic, (**c**) 500 K in the smectic-A phase, and (**d**) 600 K isotropic liquid. Reference slopes of 1, indicated as small triangles, help to identify the linear diffusive regime. The colors signify the dimension (red: *x*, blue: *y*, black: *z*); the solid lines are for the long cations and the dotted lines are for the nitrate anions. The most distinctive difference is the divergence of the *z*-direction from the *x*- and *y*-directions in the smectic-A phase, showing that transport within a layer is much simpler than *through* the layers. (Notes: (**c**) The *x*- and *y*-directions are nearly overlapping. (**d**) All directions nearly collapse, indicating an isotropic phase.)

Replace

**Figure 8:**
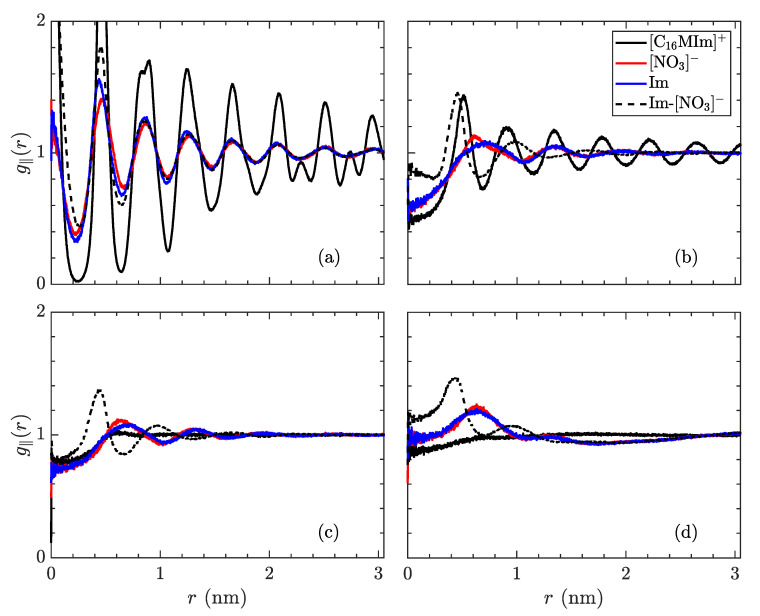
In-plane radial distribution function, $g_{\|}\left(r\right)$ of the species in [C_16_MIm][NO_3_], trajectory-averaged over the last 200 ns, at temperatures of (**a**) 300 K in the crystalline state, (**b**) 400 K just before the transition to the smectic-A state, (**c**) 425 K in the smectic-A phase, and (**d**) 600 K isotropic liquid. The solid lines signify distribution of a species relative to its own type (black: [C_16_MIm]^+^, red: [NO_3_]^−^, blue: imidazolyl ring) while the dotted line denotes placement of [NO_3_]^−^ ions in relation to imidazolyl rings. The first phase transition is characterized by the alkyl tails losing almost all order within the xy-plane. The phase transition to the isotropic liquid loses almost all structure, except for pairing of the opposite charges.

with

**Figure 8:**
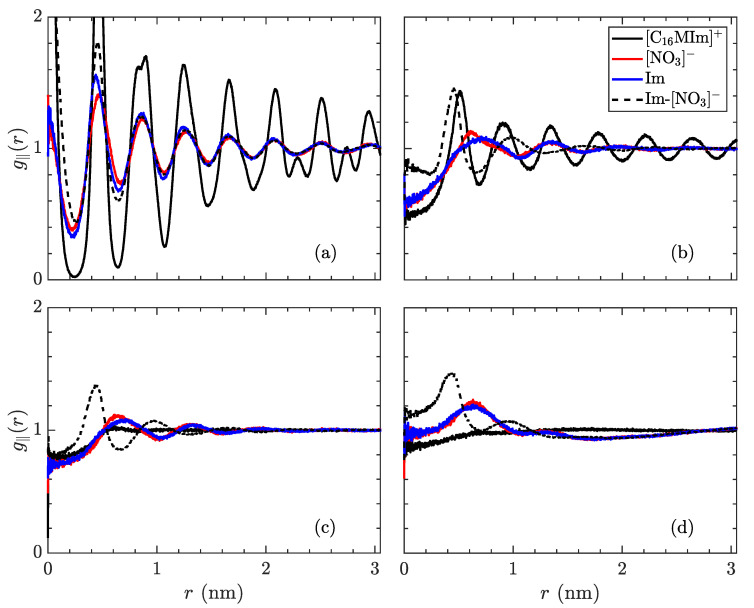
In-plane radial distribution function, $g_{\|}\left(r\right)$ of the species in [C_14_MIm][NO_3_], trajectory-averaged over the last 200 ns, at temperatures of (**a**) 400 K in the crystalline state, (**b**) 450 K just before the transition to the smectic-A state, (**c**) 500 K in the smectic-A phase, and (**d**) 600 K isotropic liquid. The solid lines signify the distribution of a species relative to its own type (black: [C_14_MIm]^+^, red: [NO_3_]^−^, blue: imidazolyl ring) while the dotted line denotes placement of [NO_3_]^−^ ions in relation to imidazolyl rings. From (**a**) to (**b**), a significant decrease in order of tail groups is observed, with the loss of secondary peaks, while the correlations also decay slightly more quickly as a function of distance. The first phase transition is characterized by the alkyl tails losing almost all order within the xy-plane. The phase transition to the isotropic liquid loses almost all structure, except for pairing of the opposite charges.

The authors regret any inconvenience these changes cause to readers, but seek to correct the record in the name of accurate and reproducible science.
